# Autonomous password generation and setting system with cosmic coding and transfer (COSMOCAT) and cosmic time calibrator (CTC)

**DOI:** 10.1038/s41598-025-87007-6

**Published:** 2025-02-13

**Authors:** Hiroyuki K. M. Tanaka, Elena Cantoni, Giancarlo Cerretto, Alan Duffy, Marko Holma, Shanti Krishnan, László Oláh, Marco Sellone, Sara Steigerwald, Dezső Varga

**Affiliations:** 1 University of Tokyo, Tokyo, Japan; 2 International Virtual Muography Institute (VMI), Global, Tokyo, Japan; 3 INRIM (Istituto Nazionale di Ricerca Metrologica), Torino, Italy; 4 Swinburne University of Technology, Melbourne, Australia; 5 mDetect, Melbourne, Australia; 6 Muon Solutions Oy, Oulu, Finland; 7 University of Oulu, Oulu, Finland; 8 HUN-REN Wigner Research Centre for Physics, Budapest, Hungary

**Keywords:** Electrical and electronic engineering, Engineering, Physics, Particle physics, Experimental particle physics, Mathematics and computing, Computer science, Information technology

## Abstract

As wireless sensor networks (WSNs) with Internet of Things (IoT) devices become increasingly widespread and more complex, the threat of cyber-attacks is also increasing. One of the most common ways WSNs can be hijacked is when passwords/IDs are leaked. If the passwords do not frequently change, it is easier for the system to be compromised. However, many organizations and individuals retain old passwords to avoid the hassle and challenge of continually remembering and managing new passwords. COSMO-PASS is a new technique that combines COSMOCAT and CTC to enable hardware-level protection of the WSN nodes. It removes the inconvenience of having its users create, remember, and change multiple passwords. Based on the test experiments and simulations with a 10^2^-cm^2^-sized (a smartphone-sized) detector, 6–7-digit passwords are automatically generated and transferred to the sensor node within the time range from 1 s to 1 min, depending on the nodal distance (10–50 cm). Consequently, it is confirmed that automatically generated and frequent password updates are possible with COSMO-PASS, which will effectively protect the data and network. Although applications of COSMO-PASS are limited to a short range, since users do not have to know or physically input the password to their system, the phishing risk is greatly mitigated. It is anticipated that the enhanced security level capabilities of COSMO-PASS can easily be applied to the next generation of secured short-haul wireless sensor networks to achieve the realization of safer and smarter communities.

## Introduction

The need for more features and flexibility in personal area networks (PANs) continues to increase as modern communities continue to integrate the concept of the “ubiquitous world” (also called ubiquitous computing systems) more fully into daily life. One of the key technologies essential for realizing the concept of a ubiquitous world is the ubiquitous sensor network (USN), which aims to make services and communication as accessible as possible. Large-scale communication infrastructure is not required for a USN; instead, this network spontaneously creates new functions and services based on information collected through machine-to-machine (M2M) communication from many small sensor devices installed in several locations. USN sensor (or IoT) devices have built-in wireless equipment for near-field communication (NFC), allowing devices to communicate directly with each other on an ad-hoc basis to form a network and collect data observed by sensors by using multi-hop routing. Examples of potential applications of USNs include wildfire outbreak prediction^1^, home security, and building automation systems (BAS). Conventional sensor networks have strong drawbacks in comparison. For example, sensor networks that predict landslides by monitoring ground displacement in hazardous river areas via dedicated lines already exist^2,3^; however, due to installation costs, these networks have only been able to detect localized events. On the other hand, USN, which consists of compact wireless sensor devices and ad-hoc networking, improves installation flexibility, thereby making it more practical and economical to increase the number of sensor nodes in a network. Beyond traditional applications, USN has the potential to monitor other completely new applications indirectly. For example, by measuring the speed distribution of the windscreen wiper movements of a number of driving cars within a given area, an estimation of the rainfall amount can be made without requiring the installation of a number of dedicated rain gauges. This approach, which integrates various types of information, is one of the strongest advantages of USN over conventional sensor networks.

However, with so many devices interconnected in a large network, USN is more vulnerable to attack from unauthorized third parties than conventional networks, which (by gaining access to even one device) could remotely hijack the entire system^4^. For example, in the case of BAS, by taking control over a single device, an attacker can gain access to vital data such as a building’s air conditioning and ventilation system, blueprints of floors and roofs, and even water pipe diagrams. It is also possible to shut down the server from a remote location by turning off one section of the system, like the air-conditioning system. In other words, if there is even one vulnerable terminal or access point, the entire system could be threatened or controlled from a remote location. The easiest way for outsiders to break into USN access would be through wireless connections, which would be popular to use for USN since wireless systems are more practical and flexible than wired systems. There are several vulnerabilities in wireless communication which could be exploited. If the SSID and network security key are leaked to a third party, they can connect to the wireless LAN. Currently, there is a practice among criminal hackers called “wardriving”, in which searches for vulnerable access points around a city are conducted while driving in cars along city streets^5^.

One of the biggest threats for WSNs is the presence of third parties who could infiltrate the system to inject malicious data into the network intentionally. Such an intrusion into sensor terminals usually takes place after acquiring leaked passwords and ID lists (including security keys). It is impractical to implement a brute-force attack to enter such terminals since there is usually a security setting that will shut down or lock the system after a certain number of incorrect password inputs. As the number of terminals increases, the user IDs and passwords that need to be managed inflate dramatically. Additionally, these passwords should be frequently changed since the threat of cyber-attacks on the computer security system originates in the leakage of such expired passwords. Under these circumstances, the United Kingdom’s National Cyber Security Centre (NCSC) strongly promotes the idea of finding new ways to release users from such password overload issues. The NCSC is working to reduce organizations’ reliance on this policy of requiring users to remember large numbers of complex passwords^6^. However, it is not trivial to establish a common security infrastructure to distribute the passwords shared among all sensor nodes (terminals), and often, there are no procedures to change shared passwords frequently (which makes these terminals more difficult to crack). COSMO-PASS technology has the potential to solve this fundamental challenge.

The COSMO-PASS system is unique from most data security systems in that it relies on data that are difficult to generate artificially, but it does not rely on the physical transfer of mathematically derived passwords. By detecting the arrival times of cosmic-ray muons, a naturally occurring phenomenon that can be used to generate true random number (TRN) sequences, with a muon detector, single-use timestamps can be generated and utilized as pre-shared passwords, and this technique acts like a one-time pad. The key features of COSMO-PASS are (A) the passwords are relatively frequently updated, (B) one password is used only once to log into the terminal, and it is updated immediately after login, (C) individuals, including the terminal users, do not have to know or have access to the passwords, (D) the passwords consist of naturally generated TRNs, and (E) the passwords are transferred between nodes wirelessly without using information traffic. These features make it impossible for a third party to crack the terminal from a remote location.

In order to apply TRNs for passwords/common keys, at least two TRN numerical sequences are independently needed for the terminal (receiver) and the user (sender). The current world record of TRN generation speed is 250 trillion TRNs per second^7^ However, these TRNs cannot be duplicated in different locations without first being copied and transferred. For this process, either a physical or cyber information exchange (e.g., bringing a USB memory from one terminal to another or via Wi-Fi) is required. As long as there is a physical/cyber information exchange, there is always a loophole that could be exploited by others to steal/crack the data, which strongly increases its vulnerability. Utilizing the pulse height distribution, or arrival time distribution of cosmic-ray muons, has long been considered an ideal source for TRNs^8^. Most of the primary cosmic rays are galactomagnetically trapped inside the Milky Way Galaxy for millions of years before leaking to our solar system and thus, the arrival time of cosmic rays to the Earth is totally random in our daily life time scale. However, there has been no way to deliver these TRNs to different locations without a physical/cyber information exchange until the emergence of the cosmic coding and transfer (COSMOCAT) technique. COSMOCAT^9^ and COSMOCAT storage (COSMOCATS)^10^ have shown their potential to drastically enhance the security of USN applications, such as BAS^9^, wireless power transfer (WPT)^9^, and digital signing and authentication services^10^. If the distance between the sender sensor and the receiver sensor is less than 10 m, and since the time required for cosmic muons to travel this distance is less than 100 ns, the arrival times can be approximated as nearly simultaneous for the purposes of timestamping. However, in order to independently acquire nearly identical timestamps, the sender and receiver sensors must be synchronized with great accuracy. Established COSMOCAT techniques that utilize an external reference time input, such as GPS-DO and UTC^11^, or wiring, can fulfill this purpose. However, such external reference time inputs are often impractical due to the additional costs of expensive devices and installation/maintenance. Moreover, GPS-DO cannot be used indoors or underground. For these reasons, applying GPS-based COSMOCAT to WSNs, including USN, has been practically challenging. For this reason, such GPS-based COSMOCAT had a strong limitation in application to WSN including USN. The basic concept of COSMO-PASS eliminates such restrictions.

COSMO-PASS utilizes the features of two newly developed techniques, cosmic coding and transfer (COSMOCAT) and the cosmic time calibrator (CTC)^12–14^ to share passwords between the sender sensor and the receiver sensor without the need to physically transfer these passwords. COSMOCAT utilizes muons—relativistic and highly penetrating elementary particles that arrive at arbitrary intervals and are ubiquitous across every part of the Earth’s surface. These particles can penetrate up to kilometers into solid or liquid media. COSMOCAT harnesses this property to generate true random number timestamps, which can be used for USN passwords. CTC, the technique that uses muons to synchronize clocks in indoor and underground environments, synchronizes the time between the sender sensor and the receiver sensor so that issued random timestamps can be implicitly shared between the sender sensor and the receiver sensor. Therefore, COSMO-PASS is effectively a combination of these techniques (“COSMOCAT + CTC = COSMO-PASS”), and operates as follows: (1) a password (common key) is generated, (2) this password (common key) is replaced with a new password (common key) every time a new muon arrives (during the Holdover Mode), (3) if the local clock has drifted, CTC calibration is applied, using previously generated passwords (during the CTC-locking mode), and (4) upon completion of the calibration, the process returns to step 2. In this paper, the principles, workflow, and limitations of COSMO-PASS will be thoroughly described, alongside experimental test results. Applications of COSMO-PASS will also be discussed.

This paper’s organizational structure is as follows. In the Results section, after a brief description of the COSMO-PASS principle, the COSMO-PASS automatic password generation and update rate are theoretically analyzed based on the zenith-angular cosmic-ray muon flux. Then, the apparatus used for COSMO-PASS consisting of two components, the COSMOCAT unit and the CTC unit, is described. Subsequently, the two COSMO-PASS operational modes, the CTC-Locked Mode and the CTC-Locking Mode, are described and examined based on experimental results for the actual password generation and update rate. Based on these results, the pros and cons of the COSMO-PASS, along with its potential applications, are outlined in the Discussion section. Lastly, the technical performance of COSMO-PASS is compared with other established authentication techniques.

## Results

### Principles

In the COSMO-PASS system, each sensor node is associated with a detector, referred to as a COSMOCAT detector, which can act both as the sender and the receiver. A key feature of COSMO-PASS is that it allows users to wirelessly generate two identical TRNs without physical data transfer for the authentication process. As previously mentioned, COSMO-PASS is based on the concept of the COSMOCAT technique^9^, which can independently generate two or more identical TRNs independently. To our knowledge, COSMOCAT is the only known technique capable of generating the same TRNs independently in different locations. If we split the signal output from one TRN generator, we can generate two or more identical TRNs; however, since these numerical sequences are merely duplicated from a single TRN source and not independently obtained (i.e., since these numerical sequences do not qualify as being), they cannot be used for securing the operating system of a terminal. Obviously, this method of simply copying a TRN sequence requires physical access to the original, significantly increasing vulnerability. COSMO-PASS was designed to overcome these problems.

### Password update rate

In this discussion, it is assumed that the size and the signal-to-noise ratio of the detectors associated with the sensor nodes are uniform. The following is a description of how the detectors cooperate within the WSN. In this scenario, Detector1 serves as the detector used for setting the passwords, while Detector2 is used for logging into the terminal; however, all COSMOCAT detectors in the WSN can perform either function as required. Detector1, a COSMOCAT detector within the network, is associated with the WSN sensor (Sensor1) and generates TRN passwords at a rate of *NR*^− 1^ Hz, where *N* is the muon rate and *R* is the signal-to-background noise ratio.

*N* is determined by integrating the zenith-angular dependence of the open-sky cosmic-ray muon spectrum^15^, given by:

1 $$dI/dEd\Omega \approx {\text{ }}0.{\text{14}}{E^{ - {\text{2}}.{\text{7}}}}[{\text{1}}/({\text{1}}+{\text{1}}.{\text{1}}E{\text{cos}}\theta /{\text{115 GeV}})+0.0{\text{54}}/({\text{1}}+{\text{1}}.{\text{1}}E{\text{cos}}\theta /{\text{85}}0{\text{ GeV}})]{\text{ c}}{{\text{m}}^{ - {\text{2}}}}{{\text{s}}^{ - {\text{1}}}}{\text{s}}{{\text{r}}^{ - {\text{1}}}}{\text{Ge}}{{\text{V}}^{ - {\text{1}}}},$$

over the solid angle (*Ω*) formed by Detector1 and Detector2. Equation (1) indicates the analytical expression of the muon’s energy-flux relation that can be reasonably derived from variations in flux with depth under rock by assuming the flux varies smoothly with energy. 115 s *q* GeV and 850 s *q* GeV in the first term and the second term of Eq. (1) are respectively the critical energies for muons (which are introduced to express the decay probability) that are derived from the decay of pions and kaons. Other parameters are given by fitting measurement results. More detailed description about this analytical expression can be found in Adair and Kasha (1977)^16^.

**Fig. 1:**
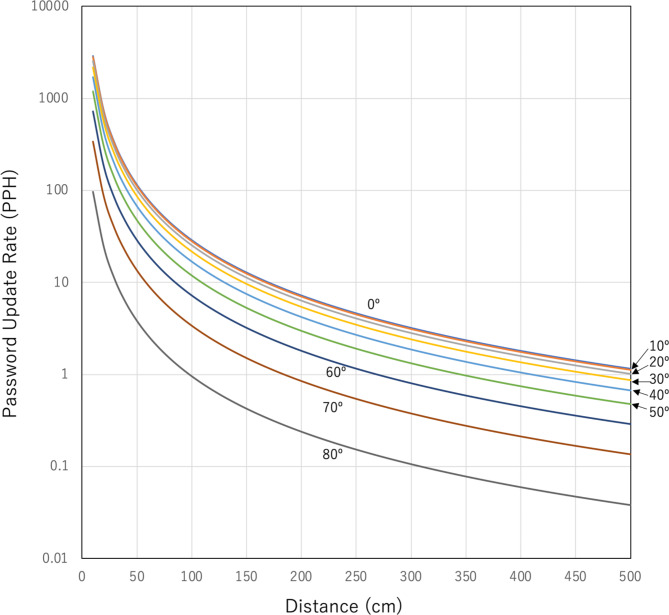
Password update rate in units of password updates per hour (PPH). The values expected values using a 10^2^-cm^2^ detector are shown for various zenith angles (0°-80°) formed between Detector1 and Detector2.

If the size of the effective active area for muon detection (the size of the active area projected onto the plane perpendicular to the line connecting the centers of Detector1 and Detector2) is fixed, the solid angle depends only on the distance (*D*) between the detectors. When *D*^2^ is much greater than the size of the effective active area for muon detection, *N* can be derived by integrating Eq. (1) over the zenith and azimuth angular range of:

2a $$\phi ={\text{ta}}{{\text{n}}^{ - {\text{1}}}}\left( {X/D} \right),$$

2b $$\theta ={\text{ta}}{{\text{n}}^{ - {\text{1}}}}\left( {Y/D} \right),$$

resulting in:

3 $$N(\theta )=\mathop \smallint \limits_{0}^{\phi } \mathop \smallint \limits_{0}^{\theta } \mathop \smallint \limits_{{{E_c}}}^{\infty } IdEd\theta d\phi ,$$

where *X* and *Y* represent the lengths of the detector in the polar and azimuthal directions, respectively. Figure 1 illustrates the calculation results of Eq. (3) for an effective active area of 10^2^ cm^2^. Table 1 summarizes the relationship between the muon’s arriving zenith angle and the required distances between Detector1 and Detector2 required to update the password in intervals of two seconds, one minute, and one hour. Considering the significantly low horizontal muon flux, it is unrealistic to utilize the muons arriving at zenith angles between 80° and 90° for COSMO-PASS automatic password generation and update and thus, direct communication between the horizontally configurated IoT appliances was not considered in this work (see more detail in discussion section.)

**Table 1:** Relationship between the muon’s arriving zenith angle (in degrees), the distance between Detector1 and Detector2 (in centimeters), and the password update rate, expressed as more than 0.5 passwords per second (PPS), 1 password per minute (PPM), and 1 password per hour (PPH).

	>0.5 PPS	1 PPM	1 PPH
Zenith angle (º)			
0	< 10 cm	70 cm	> 500 cm
40	< 10 cm	50 cm	420 cm
50	< 10 cm	45 cm	350 cm
60	< 10 cm	40 cm	270 cm
80	–	15 cm	100 cm

In *T* seconds, Detector1 generates *TNR*^− 1^ timestamps, which are directly used to update the terminal’s passwords at a rate of *NR*^− 1^ Hz. Similarly, another COSMOCAT detector (Detector2), associated with another WSN sensor (Sensor2), independently generates *TNR*^− 1^ timestamps independently from Detector1. Ideally, *TNW* timestamps out of *TNR*^− 1^ timestamps should match those generated by Detector1, as they originate from the same muons passing through both detectors. Here, *W* represents the solid angle formed between Detector1 and Detector2. Detector2 then proceeds to decode the transferred data by matching the passwords generated by Detector1 with those generated by Detector2 at a rate of *TNR*^− 1^ Hz. Since *TNR*^− 1^ > *TNW*, Detector2 must execute the matching process multiple times to log into the terminal.

The password update rate is proportional to the detector size. If the detector size is comparable to that of a smartphone (~ 10^2^ cm^2^) or a laptop PC (~ 10^3^ cm^2^), the password update rates are ~ 1×*D* Hz m^− 2^ or ~ 10×*D* Hz m^− 2^, respectively, for a nearly vertical arrangement, where *D* [m] is the distance between the detectors. As discussed in subsequent sections, the practically acquirable number of digits per event is 6–7.

### Apparatus

**Fig. 2:**
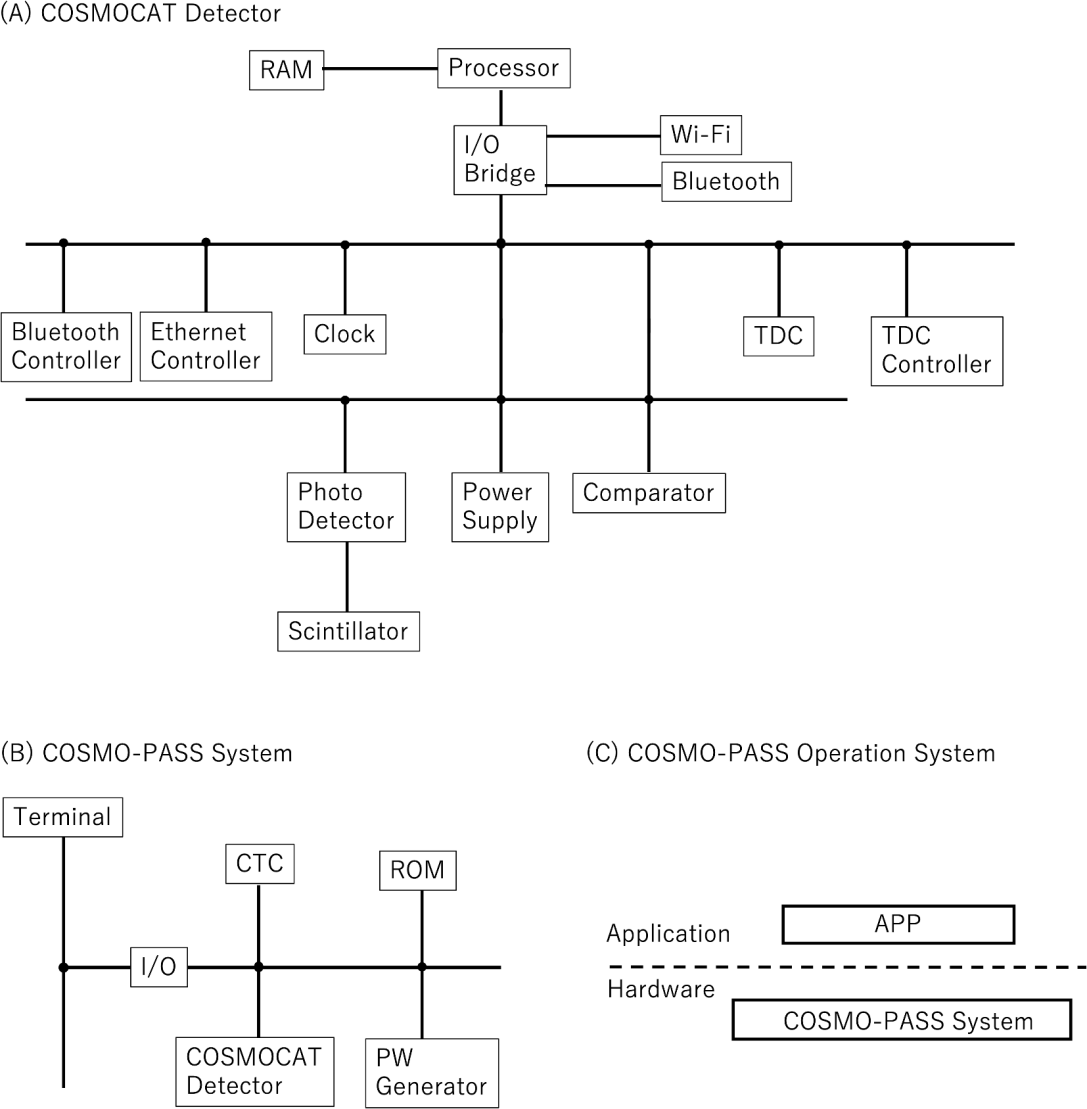
COSMO-PASS system. The components of the COSMOCAT detector (**A**), the COSMO-PASS system (**B**), and the COSMO-PASS operating system (**C**) are shown.

The current COSMOCAT detector (Fig. 2A) consists of a plastic scintillator sheet (ELJEN 200) connected with a photodetector (Hamamatsu R7724) via an acrylic lightguide, power supply, comparator, Ethernet, Wi-Fi, and NFC controllers, clock (oven-controlled crystal oscillator (OCXO)), time to digital converter (TDC) (ScioSense GPX-2), TDC controller, processor (complex programmable logic device (CPLD)), random access memory (RAM), and I/O bridge for NFC, Wi-Fi, and Ethernet. The COSMO-PASS system (Fig. 2B) consists of a COSMOCAT detector, CTC, read-only memory (ROM), and Password Generator (PW Generator). Cosmic-ray muons arrive at the plastic scintillator sheet and are detected by the photodetector. The signals outputted from the photodetector are discriminated with a comparator, and fed to the TDC. On the other hand, 10-MHz LVTTL (level transistor-transistor logic) pulses generated by the clock are fed to the TDC. The TDC counts the number of pulses from the clock and simultaneously measures the relative muon arrival time measured from the clock pulse. The clock time and the muon arrival time are integrated with CPLD (the integration process is described in the COSMOCAT Unit subsection in detail). This integrated time is handed to COSMO-PASS to filter it (the process is described in the COSMOCAT Unit subsection in detail). This synthesized time is also used for the clock calibration with the CTC technique (the process is described in the CTC Unit subsection and the CTC-Disciplined Mode subsection in detail) to synchronize the counterpart COSMOCAT detector. The COSMO-PASS application is installed on the terminal to operate the COSMO-PASS system (Fig. 2C).

### COSMOCAT unit

This section describes the COSMO-PASS device concept, which consists of the COSMOCAT Unit and the CTC Unit. The following subsections introduce details on their concurrent operation.

Detector1’s COSMOCAT Unit includes a power supply, a scintillation detector consisting of a photodetector (PD) connected to a plastic scintillator sheet via an acrylic light guide or wavelength-shifting (WLS) fibers, and associated electronics. The associated electronics feature an oven-controlled crystal oscillator 1 (OCXO1), a comparator, a time-to-digital converter (TDC), a pulse counter (scaler), a processor such as a CPU or FPGA, and an I/O bridge to Wi-Fi/BLE (Bluetooth Light Energy). The TDC measures the time interval (*DT*_*i*_, where *i* is the event number) between the moment of the PD outputs and the moment the nearest edge of the 10-MHz TTL pulses is outputted from the OCXO1. The time range of the current TDC must be sufficiently longer than 100 ns to measure the period of these 10-MHz pulses accurately. By counting the number of 10-MHz pulses (*N*_10_) and adding *t*_*i*_ to *N*_10_ × 100 ns, the muon’s time of arrival at the COSMOCAT Unit is determined. This time calculation forms the basis for generating the timestamp used in password updates. Specifically, the timestamp, calculated as *T*_0_ + *N*_10_ × 100 ns + *DT*_*i*_, is determined to update the password. Here, *T*_0_ is defined as ‘time zero,’ a parameter that can be arbitrarily set by the user to anchor the time calculations. This will be described later in more detail.

Detector2’s COSMOCAT Unit consists of the same hardware components as Detector1. Likewise, the TDC associated with Detector2 measures the time interval (*Dt*_i_) between the moment of the PD outputs and the moment when the nearest edge of the 10-MHz TTL pulses is outputted from the OCXO2. The resulting timestamp, *t*_0_ + *n*_10_ × 100 ns + *Dt*_*i*_+ *d* (*t*), is filtered to use for logging into the terminal, where *dt*(*t*) represents the relative time deviation arising from the clock drift.

### CTC unit

**Fig. 3:**
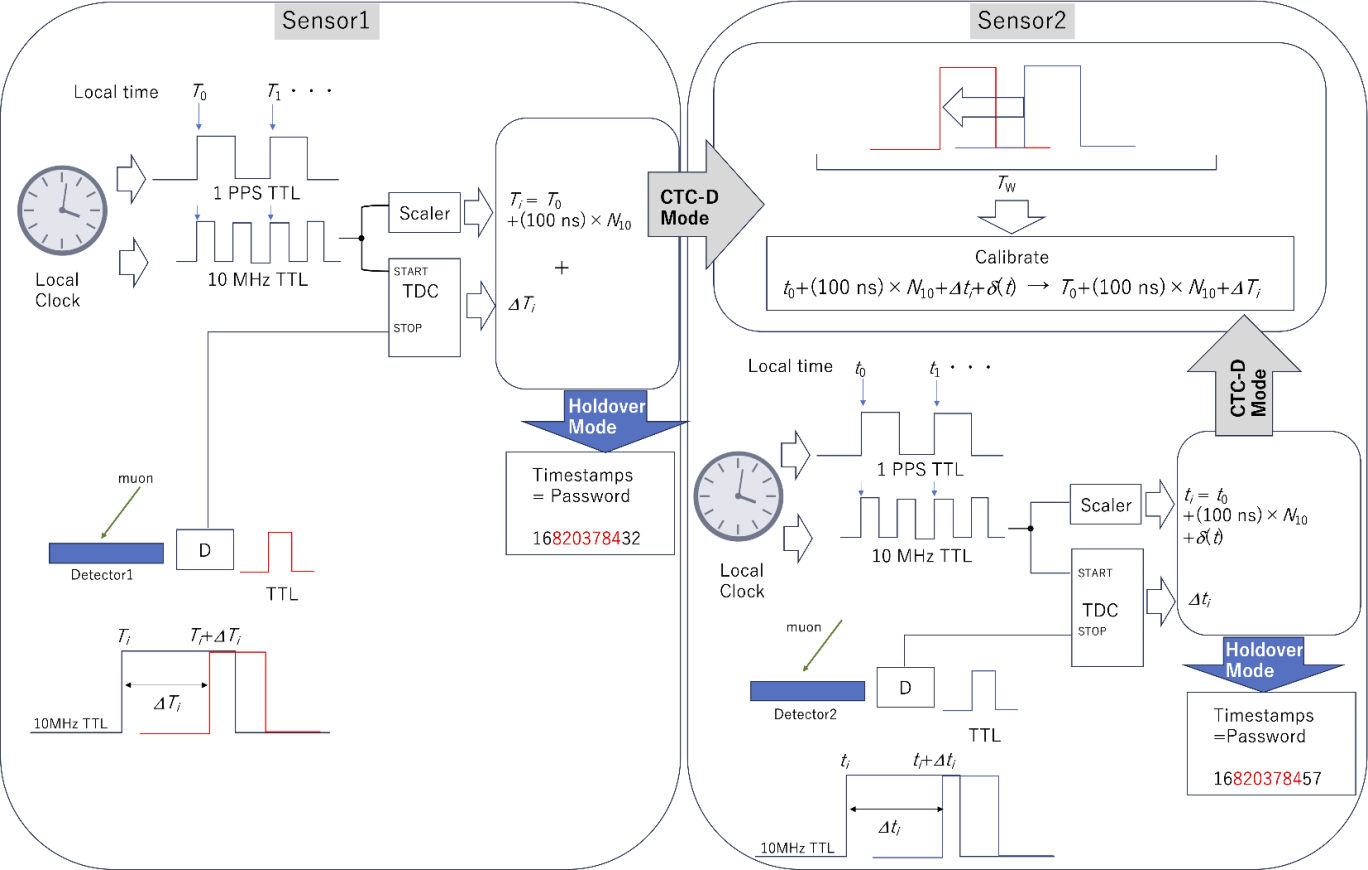
Schematic diagram of the COSMO-PASS concept. CTC-D Mode denotes the CTC-locking Mode. Holdover Mode denotes the CTC-locked mode. Red digits represent the timestamps after being filtered to generate passwords.

The CTC Unit shares the same hardware components as the COSMOCAT Unit. The time indicated by OCXO1 generally deviates from the time indicated by OCXO2 (*dt*(*t*) ≠ 0). The CTC Unit corrects this deviation. Detector2 periodically receives timestamps from Detector1 to adjust OCXO2. These timestamps are transferred via Wi-Fi/BLE from Detector1 to Detector2. The CTC Unit calculates the difference between *T*_0_ + *N*_10_ × 100 ns + *DT*_*i*_ and *t*_0_ + *n*_10_ × 100 ns + *Dt*_*i*_+*d*(*t*). Given the relatively low rate of cosmic-ray muon arrival, it is reasonable to assume that two events measured within a time window (*T*_W_), which is sufficiently narrower than the average muon arrival time window, originate from the same muon passing through both Detector1 and Detector2. Therefore, the CTC Unit corrects the OCXO2 counts by the value of (*t*_0_ + *n*_10_ × 100 ns + *Dt*_*i*_+*d*(*t*)) – (*T*_0_ + *N*_10_ × 100 ns + *DT*_*i*_). The detailed procedure of this calibration is described in the following subsections. The schematic diagram for the COSMO-PASS process is shown in Fig. 3.

### CTC-locked mode

**Fig. 4:**
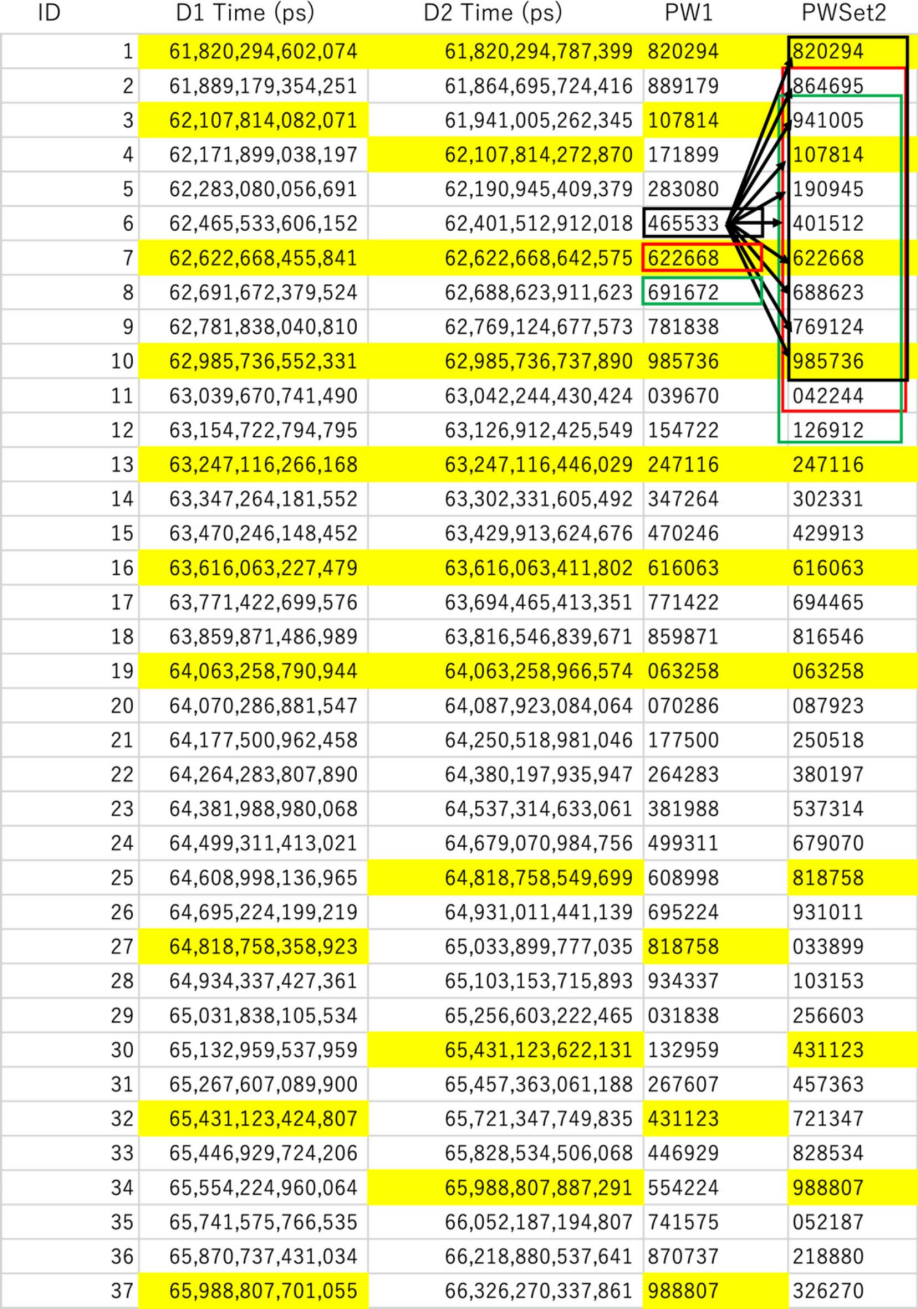
Example of the event list acquired in ~ 4 s. The first, second, third, fourth, and fifth columns respectively indicate the event ID, the timestamp generated by Detector1 (D1 Time), the timestamp generated by Detector2 (D2 Time), the filtered D1 Time (PW1), and the filtered D2 Time (PW2). The numbers highlighted with yellow markers indicate the timestamps matching within a time window of 1 ms (the second and third columns) and the corresponding filtered timestamps (the fourth and fifth columns). The black, red, and green boxes in the fifth column indicate the password set used for logging into Sensor1, protected by the passwords indicated by the boxes with corresponding colors in the fourth column.

**Fig. 5:**
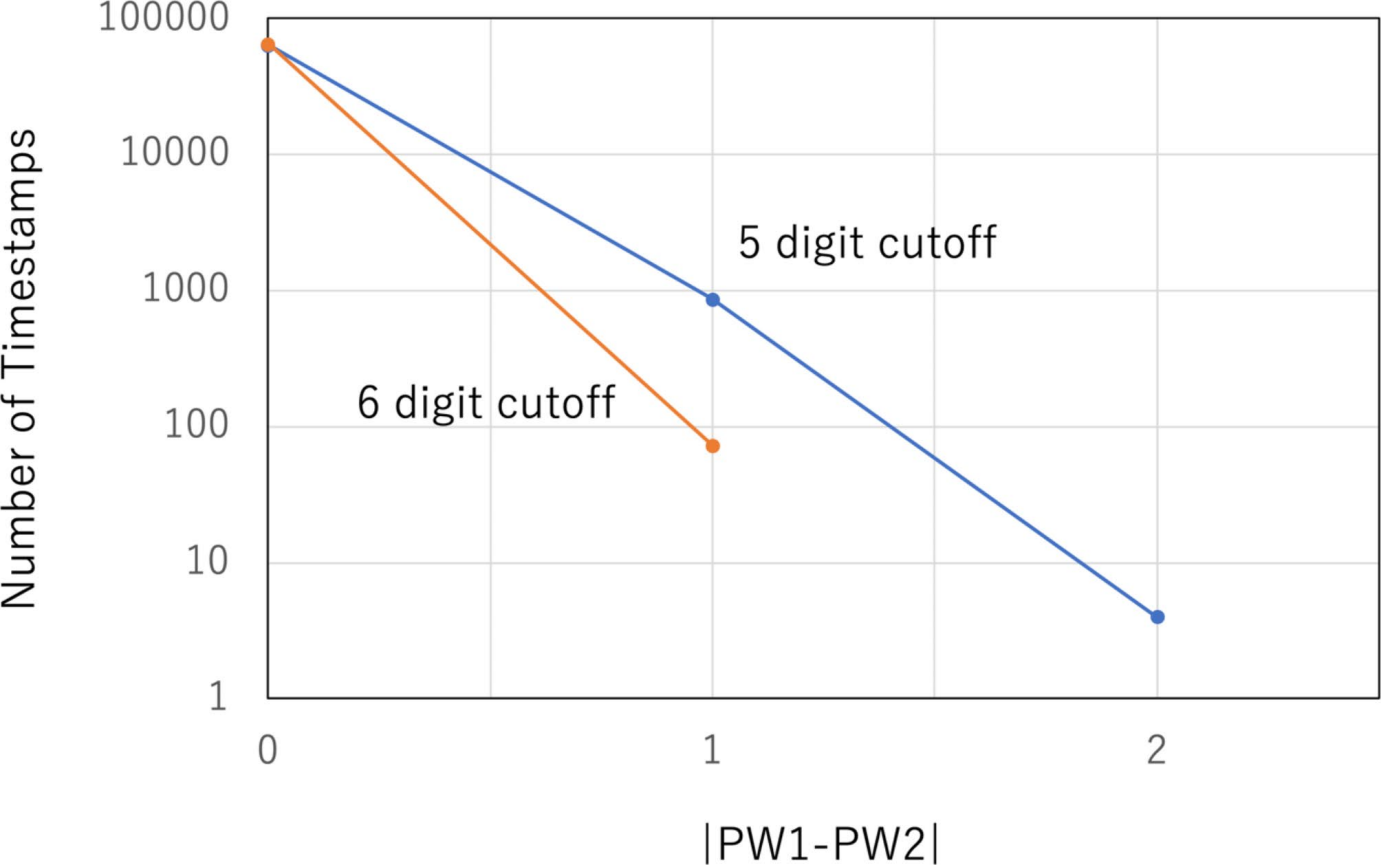
Password miss-matching rates. The number of filtered timestamps is shown as a function of the difference between PW1 and PW2 for the 5-digit cutoff (blue) and the 6-digit cutoff (orange).

This operational mode, used when the local clocks associated with the detectors are well synchronized, is called CTS-Locked Mode. With COSMOCAT, a local clock and a TDC associated with each detector generate 14-digit timestamps (in picoseconds) (shown in the second and third columns of Fig. 4) based on the moment when the signal is outputted from the detector. However, not all of these digits can be used as passwords. Figure 4 shows an example of the time-sequential list of the timestamps collected in the current work. Due to the muon rate, the first 2 digits are likely to repeat frequently. Therefore, in practice, the longest practical password that can be created within an approximate period of 10^*k*^ seconds with COSMOCAT tends to be (12 + *k*) digits long. Moreover, the last 5 digits are unlikely to match between detectors due to fluctuations caused by the detectors’ jitter and the local clocks’ granularity. Therefore, the most practically useful password length will be (7 + *k*) digits. This process of omitting the first and last digits from the timestamp is referred to as “timestamp filtering” (PW1 and PW2 in the fourth and fifth columns of Fig. 4). However, even after cutting off the last 5 digits, mismatches between the password detector and the lock detector may still occur. Figure 5 illustrates the frequency of password generation as a function of the difference between two passwords generated with the current setup for cutting off the last 5 digits and the last 6 digits. The measured mismatching rates were ~ 1% and ~ 0.1% for the last 5 digits and the last 6 digits, respectively. If these passwords are employed to secure each device, this password length is sufficient since the system can be configured to shut down or prevent users from logging in for a specific period after failing to match the correct password after approximately 10^2^ attempts, for example.

**Fig. 6:**
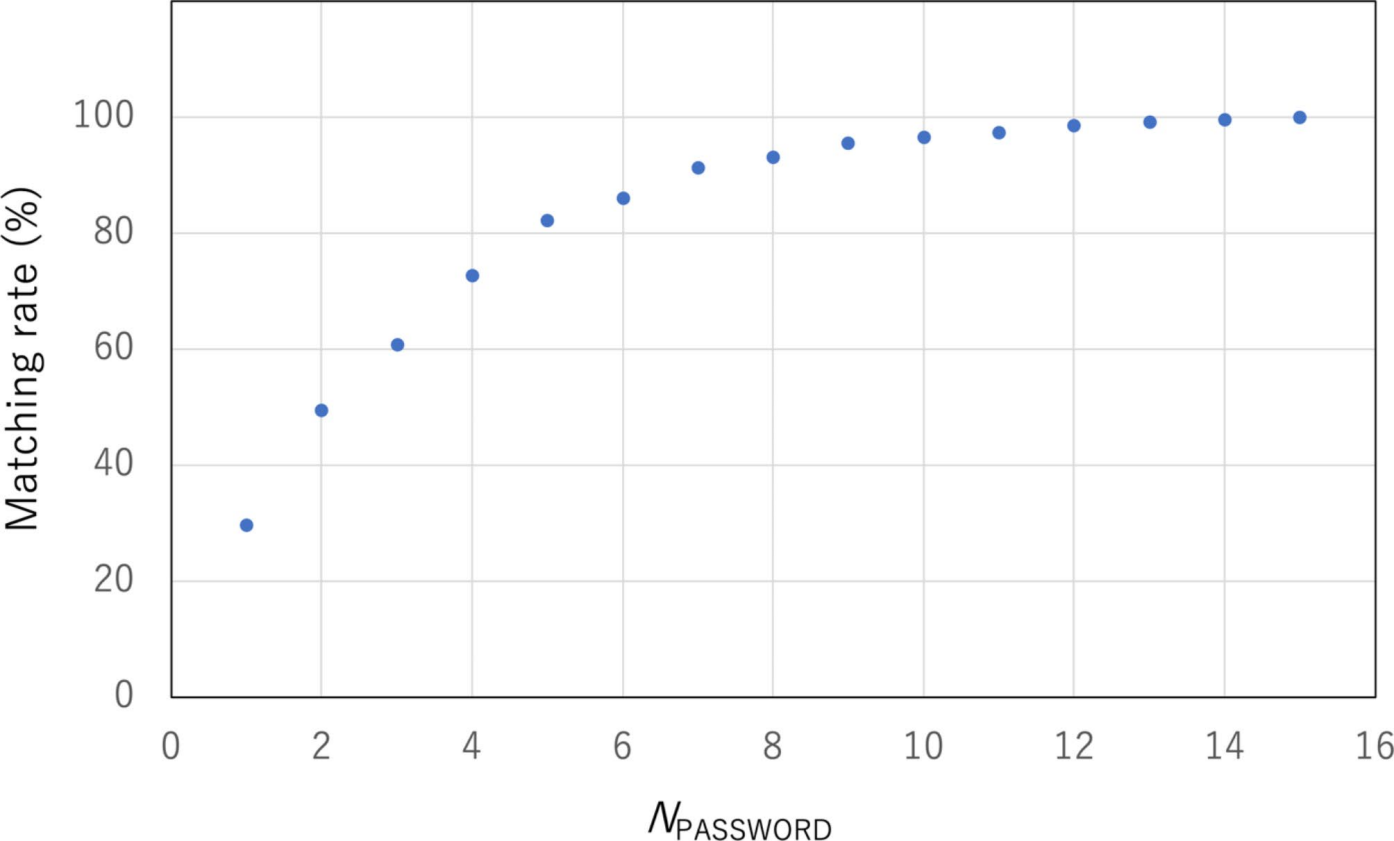
Matching rate between PW1 and PW2 as a function of the halfwidth (*N*_PASSWORD_) of the password set.

**Fig. 7:**
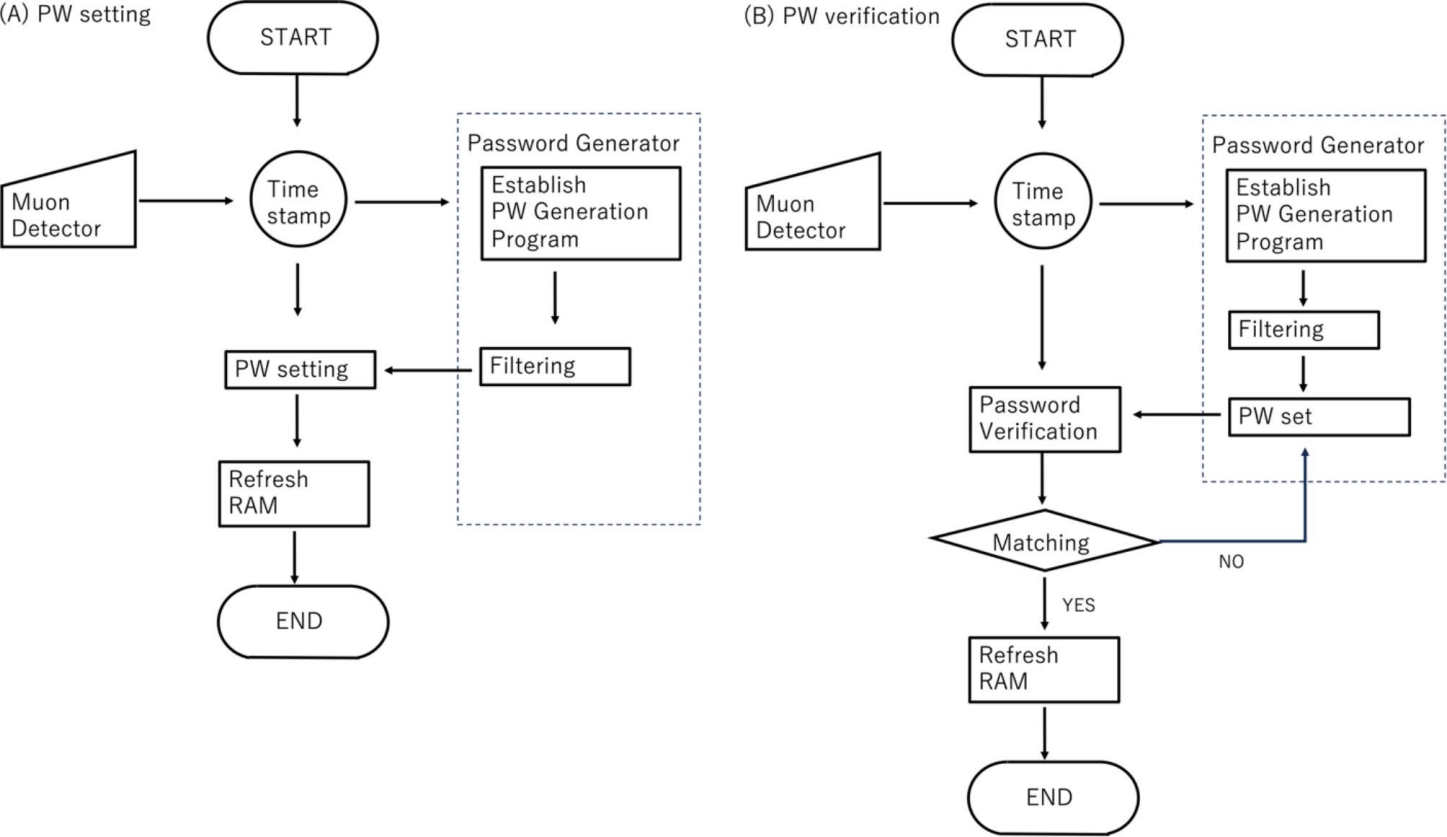
Flowcharts of the process in Holdover mode. The password setting process (**A**) and the verification process (**B**) are shown.

As previously mentioned, not every password generated by Detector2 can be used to log into Sensor1. As shown in Fig. 4, due to the detector’s efficiency and background noise, not all PW1 passwords match PW2 (unmarked PW1 and PW2 in Fig. 4). If a non-matching PW1 is set as the password for Sensor1, the user of Sensor2 cannot log into Sensor1 with PW2. To address this issue, a set of several PW2 versions, indicated by the colored boxes in the fifth column of Fig. 4, is retained in Sensor2’s random access memory (RAM) until the password verification process is completed. The halfwidth of this password set is defined as the number of passwords (*N*_PASSWORD_) generated before and after the moment when PW1 is generated. Therefore, the total width of the password set is 2*N*_PASSWORD_. In the current example shown in Figs. 4 and 5 passwords generated before and 5 passwords generated after the moment when PW1 is generated are retained in Sensor2. Sensor2 uses this password set to attempt to log into Sensor1. If Sensor1 sets PW1 with a non-matching password (unmarked PW1 in Fig. 4), the user of Sensor2 cannot log into Sensor1 even with this password set. In such cases, the user of Sensor2 must wait until the next password is set at Sensor1. These combined timestamps are used as a new password that replaces the previous one. Figure 6 shows the measured matching rate between PW1 and PW2 as a function of the halfwidth of the password set. As demonstrated in this figure, an *N*_PASSWORD_ of 5 is practically sufficient. Figure 7 shows the flowchart of the process in CTC-Locked Mode.

### CTC-locking mode

The timestamps used for password segments can be arbitrary, but they must be well synchronized between Detector1 and Detector2. When initializing the COSMO-PASS system, the users need to synchronize the clocks between Detector1 and Detector2 by using NTP server-free NTP software such as NetTime, achieving an accuracy of at least 10 ms. Should the local clock time drift, the local clocks associated with the detectors must be synchronized using the CTC technique^12^.This method operates under the assumption that both detectors are close enough for each incoming muon to pass through Detector1 and Detector2 at almost exactly the same time. The expected time difference is < 100 ns within a 30-meter distance, which falls well within the practical operational range of the COSMO-PASS system, as shown in Table 1.

**Fig. 8:**
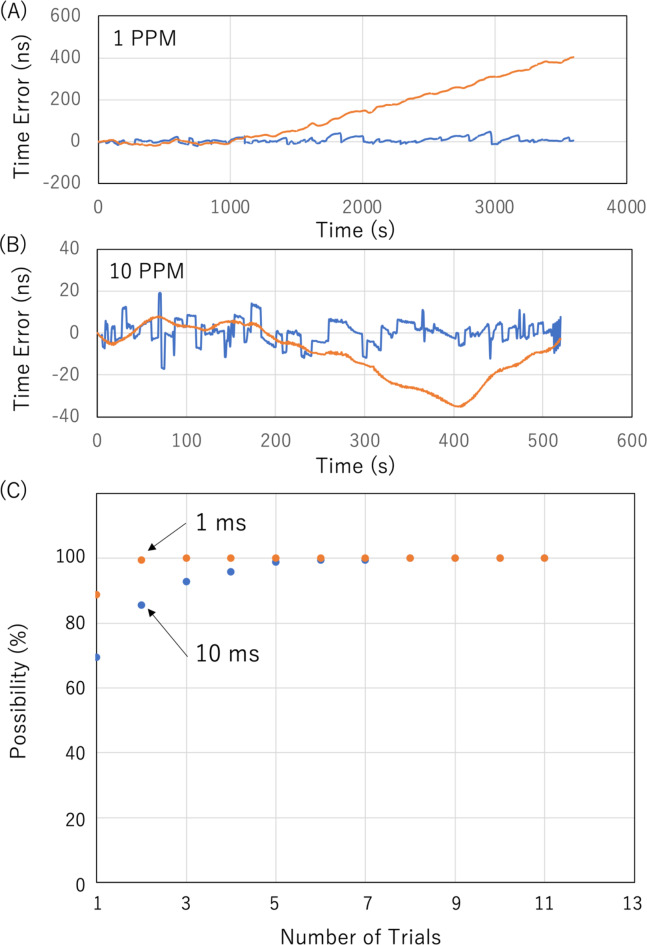
CTC results. Examples of the COSMOCAT detector’s clock timelines with (blue solid lines) and without (orange solid lines) the CTC time correction at password update rates of 1 PPM (**A**) and 10 PPM (**B**) are shown. The number of trials required to transition from the initial COSMO-PASS status to the CTC-Locking Mode. (**C**). The possibility of shifting to CTC-Locked mode, based on the number of trials needed to identify coincidence events within a 1-microsecond window, is plotted. Values are presented for the initial-stage time windows of 1 ms (orange-filled circles) and 10 ms (blue-filled circles).

**Fig. 9:**
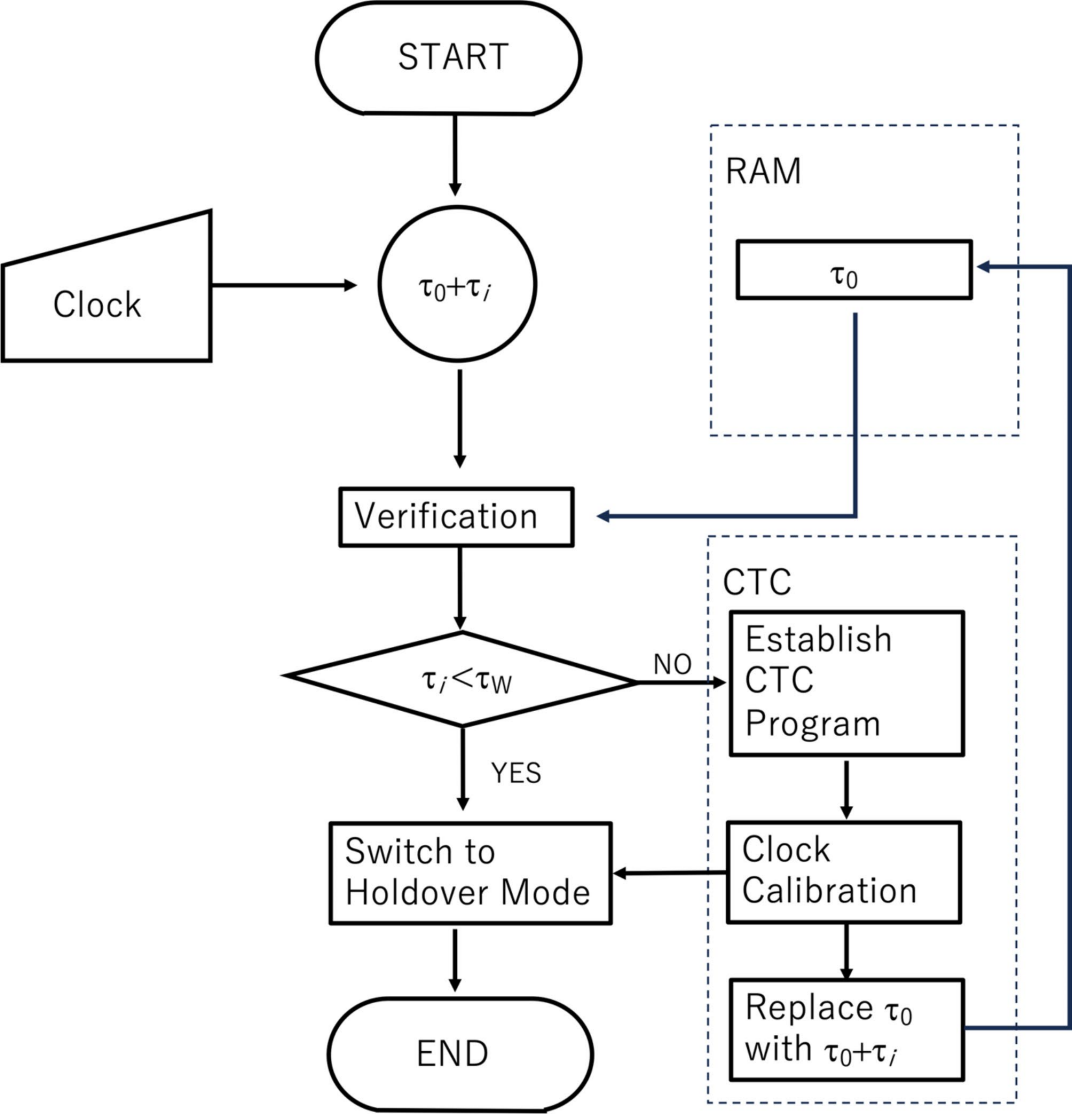
Flowchart of the clock calibration using CTC.

**Fig. 10:**
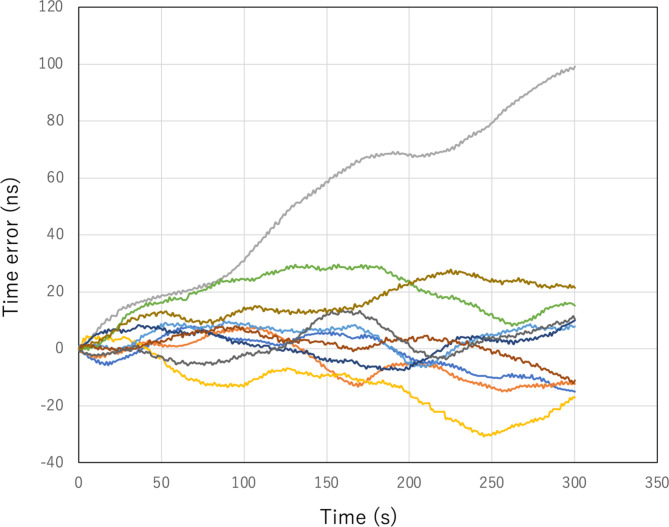
Time errors associated with OCXO, based on results from 10 independent runs.

As an example, assume a coincidence rate of *NW* ~ 1 Hz and *NR*^− 1^ ~ 4 Hz. Initially, coincidence events between Detector1 and Detector2 are searched for within a 1 ms time window (the first-stage time window). Within this window, the accidental coincidence rate is calculated as 2 × 4 × 4 × 10^− 3^ = 0.032 Hz. If this window is extended to 10 ms, the accidental coincidence rate increases to 0.32 Hz. Once coincidence events are identified, the time window is narrowed to 1 ms (the second-stage time window). If no coincidence events are observed within this narrower window for a period *T*_WAIT_ that is sufficiently longer than the average time interval *N*^− 1^*W*^− 1^ (e.g., 3 s), the window reverts to 1 ms. This process is repeated until coincidence events are identified within a 100 ns window. Figure 8A and B show examples of the COSMOCAT detector’s clock timeline with and without the CTC time correction for different password update rates: 1 PPM and 10 PPM. Due to lower CTC correction frequency, the time error with an update rate of 1 PPM (12.68 ns SD) was larger than the time error with an update rate of 10 PPM (5.43 ns SD), which was much larger than the jitter of the current detector (~ 1 ns SD). However, both of the time errors were acceptable for the current purpose. Figure 8C displays the measured number of trial cycles required to successfully find the coincidence events within a time window of 1 ms, showing initial time windows of 10 ms and 1 ms. For example, when the initial time window was set to be 1 ms, the possibility to shift from the initial status to the CTC-Locking Mode was ~ 90% at the first trial, meaning the system directly went into the CTC-Locking Mode without *T*_WAIT_. The results indicate that, on average, 0.59 and 0.12 trial cycles are respectively needed. This means that for an initial time window of 1 ms, it took ~ 0.4 s on average to initiate CTC (assuming *T*_WAIT_ = 3 s). Figure 9 shows the flowchart of the clock calibration using CTC. After this initiating process, the COSMO-PASS system is not in the CTC-Locking Mode (it reverts to CTC-Locked Mode), but this calibration is performed at regular intervals (*t*_*i*_, e.g., Holdover Mode for 1 min, and CTC-Locking Mode for 1 s) to synchronize the two detectors and to avoid errors coming from time drifting of the detector’s clock. As shown in Fig. 10, typical time errors for OCXO range from 1 ns to 10 ns per second. Therefore, the first-stage time window is set to be much narrower (e.g., 1 ms), and no waiting time (*T*_WAIT_) is required for this calibration step since the expected accidental coincidence rate is 2 × 4 × 4 × 10^− 6^ = 0.000032 Hz which is negligible. During the CTC-Locking Mode, the passwords are not updated since the timestamps are digitally exchanged via Wi-Fi, which, in principle, makes the timestamp information vulnerable to hacking. Table 2 outlines the time required for initializing the CTC Unit to find events that coincide within the 2nd *T*_W_ when *NR*^− 1^~ 4 Hz.

**Table 2:** Relationship between the muon rate (*NW*), the noise rate (*NR*^− 1^), the first-stage time window (1st *T*_W_), the second-stage time window (2nd *T*_W_), *T*_WAIT_, and the time required to initialize the CTC unit to find events that coincide within the 2nd *T*_W_.

NΩ (Hz)	NR^-1^ (Hz)	1st T_W_ (ms)	2nd T_W_ (ns)	T_WAIT_ (s)	Initialization time
1	4	1	100	3	0.4 s
0.1	4	1	100	30	18 s
0.01	4	1	1000	300	15 min
0.001	4	1	1000	3000	3.8 h

## Discussion

A potential drawback of COSMO-PASS might seem to be that anyone owning a COSMOCAT detector could log into any terminal with this system. However, it is unlikely that an unauthorized third party could gain access in this way. Each terminal’s official COSMOCAT detector is authenticated through a special initiation process (using NTP, etc., at a millisecond-level precision^17^) to establish the “first-stage time window,” which creates a unique timecode for each detector. Prior to entering CTC-disciplined Mode for synchronization, this special timecode serves as an additional password. Since the timecode used for COSMOCAT is arbitrary, it is nearly impossible for a third party to predict or calculate the exact timecode associated with a given terminal. Additionally, the time that the detector clock is set to zero usually occurs when the COSMO-PASS system is installed and activated, and users can reset this clock at their discretion.). It is thus difficult for an unauthorized third party to know or discover this timecode. Consequently, the only way a third-party attacker could possibly obtain this timecode is by physically accessing or stealing the device hardware and successfully logging into this hardware to extract this information. Therefore, with reasonable physical security measures in place, the authentication and security services provided by the COSMO-PASS system remain secure.

Recently, every sector has been enthusiastically promoting digital transformation, and a multitude of different information and technology tools have become essential to corporations. Accordingly, the number of user IDs and passwords that need to be managed for many companies and organizations has started to increase significantly^18–21^. However, as mentioned in the Introduction section, most password management policies are inadequate. Moreover, these policies are only rarely reconsidered and updated, and they tend to follow outdated advice: set up a random and complicated password that is difficult for human beings to remember. Clearly, enforcing a policy that requires users to set up and input complicated passwords is not the best solution for the users or the entire organization. If the users forget the passwords, or if their accounts are locked due to typing errors associated with their passwords, the system administrator must urgently respond to their inquiries. One strategy to alleviate this password overload, applied to the security for operating systems of terminals, is biometric (e.g., fingerprint/eye) authentication^22,23^, which is based on recognizing defects/anomalies with digital image processing; this is uniquely attributed to individuals, making it difficult for a third party to generate the authentication artificially. However, this security strategy retains privacy concerns^24,25^. Furthermore, since the fingerprint/eye data does not change over time, if these image information data are leaked or stolen, the associated terminal can be immediately compromised.

### Applications and limitations

The main drawbacks of COSMO-PASS are the limited password update rate, which depends on the cosmic-ray muon flux—a naturally limited resource—and the distance between IoT devices. Therefore, when users need to change the settings of these devices instantly (within 1 s), they must always physically approach them. However, this drawback can often be mitigated. This issue is addressed later in this subsection through the introduction of the practical application of COSMO-PASS into a home network and its performance evaluation.

Recently, the demand for networks designed specifically for homes has increased. A home network allows multiple IoT digital devices to connect and easily share various data easily within a home. Data and peripheral devices can be used more effectively as they can be accessed from anywhere within radio wave range. For the sake of simplicity, it is assumed in this discussion that all devices in the home network run on MS Windows OS in this discussion, but the same logic is applicable to other OS types. First, the users or administrators set up the COSMO-PASS system to for each terminal. Next, each COSMO-PASS detector generates a password and requests a password change. The following tasks are then implemented at each terminal when the detector requests a password change:

(A) The Local Security Authority (LSA)^26^ (the service process called sass.exe in LSA) receives the password change request from the COSMOCAT detector.
(B) The password is verified with the password filter dynamic linking library (dll) (Passfilt.dll).
(C) If successfully verified, the password is stored in the Security Account Manager (SAM).
(D) If successfully stored, the updated password is reported to Passfilt.dll, and the function PasswordChangeNotify() is called.

The processes from (A) to (B) are repeated each time the LSA detects a password change. When a user wishes to log into one of these terminals, the following tasks are implemented:

(A) The login user interface process is initiated (Logon UI).
(B) Winlogon launches the LsaLogonUser function.
(C) LSA searches for the appropriate authentication package.
(D) COSMOPASS provides the password, and Winlogon calls LsaCallAuthenticationPackage in LSASS (LSA Server Service).
(E) After authentication, Winlogon facilitates the user’s login into the terminal.

**Fig. 11:**
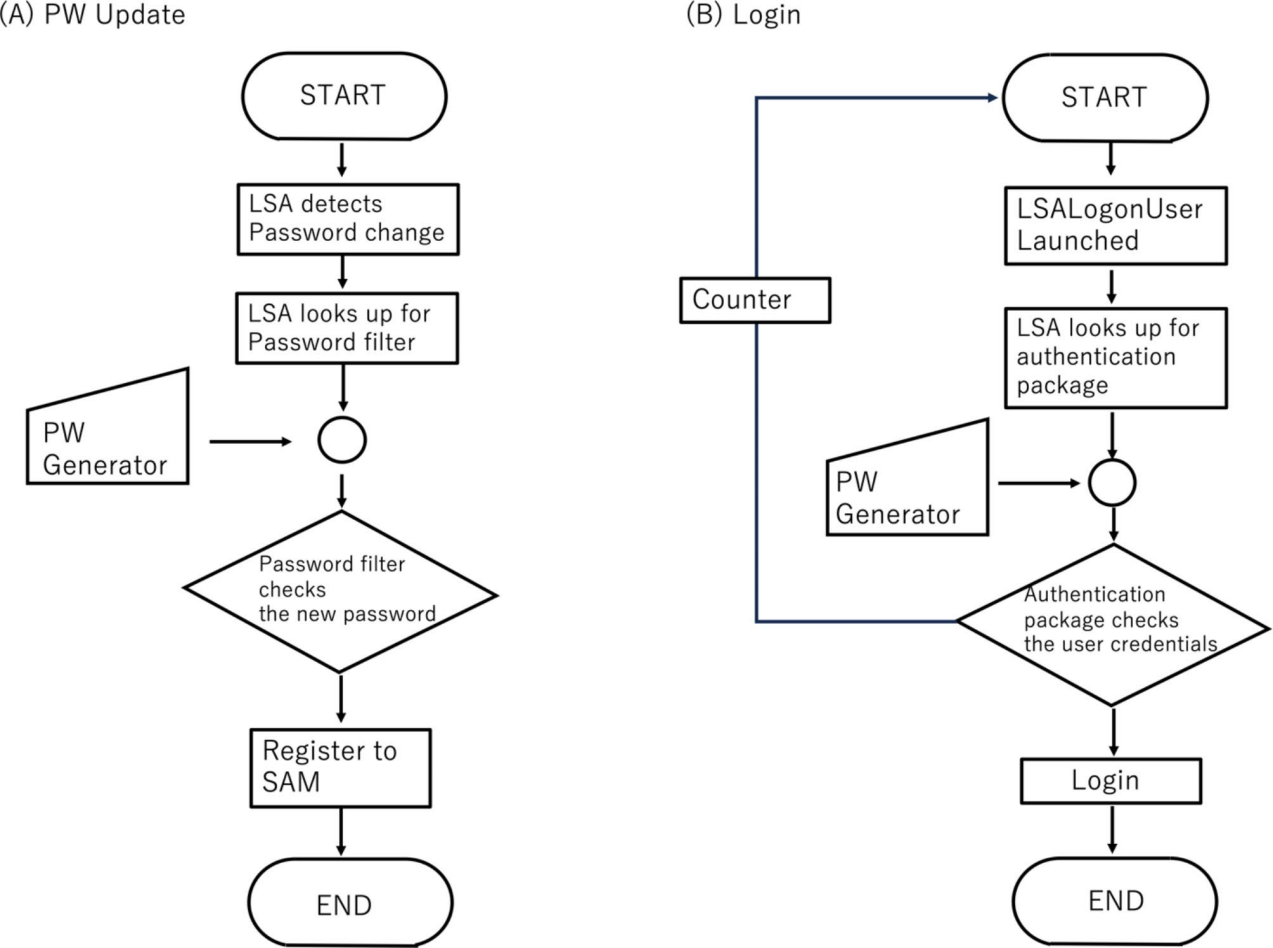
Flowcharts for the password setting. (**A**) and the login process (**B**). The counter module counts the number of incorrect password inputs to activate either the system shutdown or the system lock process.

By following this process, each device is protected by frequently updated passwords, allowing users to safely log into each device from their terminal, even without knowing the specific password. Therefore, users are not required to memorize all passwords associated with each device or frequently change them manually. The flowcharts for the password setting process and the login process are depicted in Fig. 11.

**Fig. 12:**
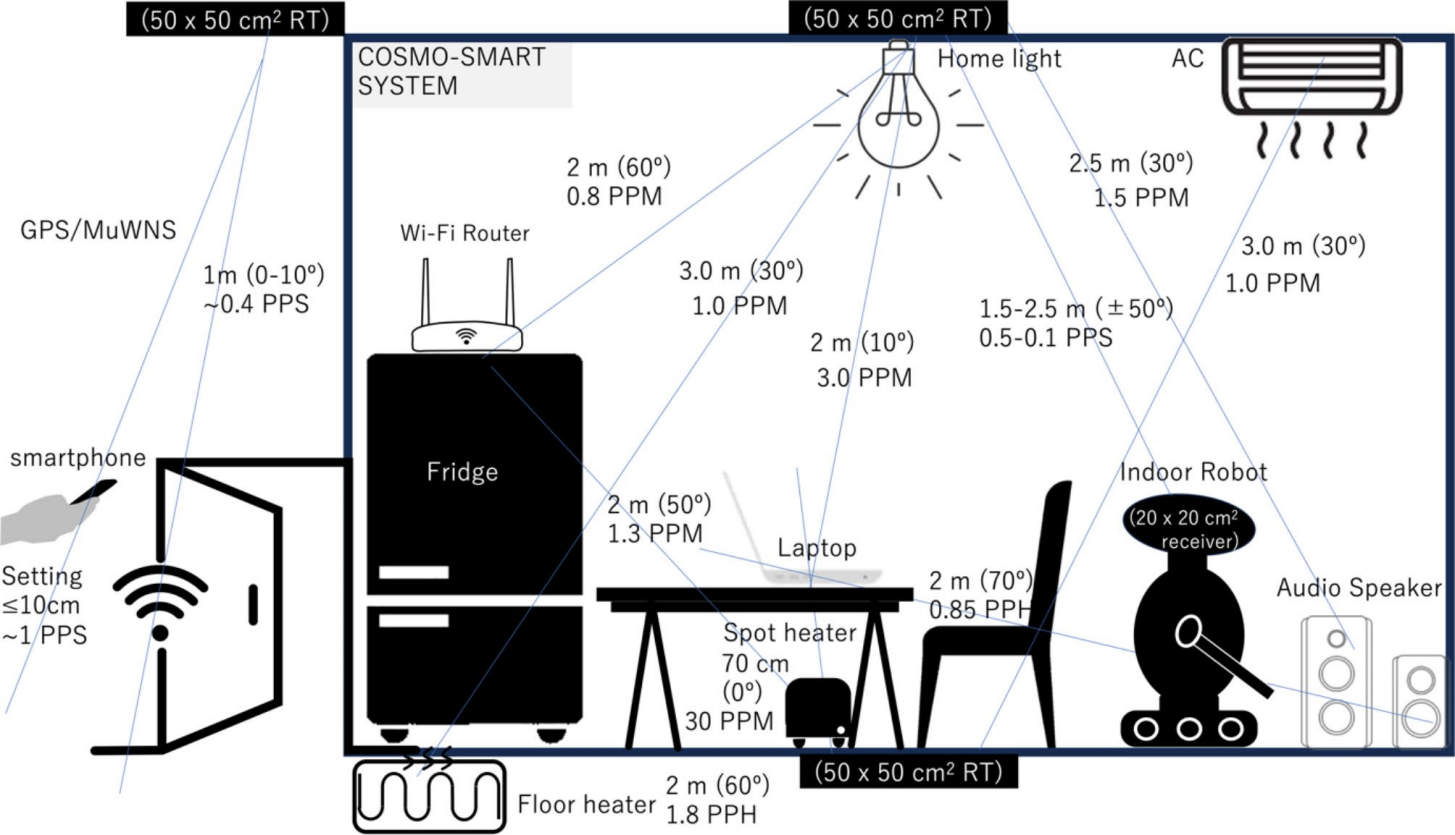
Proposed CosmoSmartAutomation system. This figure shows the typical distances and angles formed by the IoT devices, along with the password update rate in units of password per minute (PPM) and password per second (PPS). The label “RT” indicates the reference tracker used for MuWNS and COSMO-PASS. In this scenario, the reference tracker measures 50 ×  50 cm^2^ and is installed above the ceiling and underneath the floor for indoor navigation (e.g., to operate an autonomous indoor robot). The user’s approach to the door is detected with MuWNS at a cm-level accuracy, triggering a switch of the COSMO-PASS system from the Remote Mode to the Juxtapose Mode (and vice versa when the user is leaving the house). The time required for password generation and sharing can be significantly shortened within a multi-hop ad-hoc network. For example, generating a password between a laptop and an audio speaker typically takes more than one hour. However, if an air conditioner (AC) positioned between the laptop and the speaker serves as a relay station, this timeframe is reduced to less than one minute.

Figure 12 illustrates a schematic drawing of a schematic drawing of an application of COSMO-PASS to the CosmoSmartAutomation (CSA) system for a home network. A smart home industry^27–29^ is the second largest segment ($108 billion USD) while the largest segment is smart manufacturing ($119 billion USD)^29^. However, it also generates another set of security and privacy issues^28^. The CSA system protects the private data of users. In this configuration, the passwords are typically updated every ten seconds. This update rate is sufficient to protect internet-connected IOT devices from cyber attackers. However, such a relatively low password update rate may be somewhat impractical for users needing to log into their terminals frequently, as it requires tens of seconds each time they wish to change device settings. There are two options to address this issue:

1. Remote Mode: Give up the one-time pad (OTP) style authentication and use the last matched password until the next password update. In this mode, the COSMO-PASS system does not update the terminal passwords, allowing users to log into their remote terminals using the previously generated password (transferred to their terminals via information traffic such as Wi-Fi) to log into their remote terminals until it switches to the Juxtapose Mode.
2. Juxtapose Mode: Users must be physically present to log into the devices, control the devices, and retain OTP-style authentication. In this mode, users can log into their terminals using their COSMOCAT detector-embedded smartphones. Although this method may be slightly inconvenient, it ensures that the password, used only once for logging in and updated immediately afterwards, offers robust protection for IoT devices within the home network.

In practice, a hybrid of Remote Mode and Juxtapose Mode would be more practical. An example of this hybrid use of COSMO-PASS is described later.

The costs required to build a small COSMOCAT detector are reasonable. If the COSMO-PASS is attached to a smartphone (CosmoSmartPhone), the additional components needed (besides a high-end clock) include a scintillator sheet, wavelength-shifting fiber, SiPM, and comparator, costing approximately $10, $1, $20, and $2 USD respectively. The additional power consumption from these devices is less than 1 mW. All other necessary components to build COSMO-PASS, including the ps-resolving TDC (such as VL53L0X, also known as a time-of-flight/TOF sensor, which is used for laser ranging^30^), the clock, the RAM, processor, the I/O bridge (for BLE and Wi-Fi), and the power supply are already incorporated in most smartphones. However, the clocks in most smartphones are typically inexpensive quartz type of clocks, with a typical drift of ~ 10 microseconds per second. This type of quartz clock is suitable for the COSMO-PASS system by providing a 4-digit password. Therefore, the decision to incorporate an OCXO (costing $100 USD) must be carefully considered. However, more precise time synchronization capabilities will be required for smartphones in the 5G era, and accordingly, more precise clocks will likely become standard components in smartphones.

An example of this hybrid use of COSMO-PASS is described below. A smart door lock system^31,32^ could be designed using COSMO-PASS to enable users to lock and unlock the door remotely and safely via the internet. Parents would not need to provide a physical door key to their young children and could instead unlock the door remotely for them. However, if the system password were to leak, anyone could unlock the door. When authorized users (e.g., parents) are located many miles away from their home, the previously authenticated password is retained in their terminal, such as a smartphone, and is used to remotely lock and unlock the door. This password does not change during Remote Mode. As users approach their door, the system, utilizing MuWNS, detects their presence and switches COSMO-PASS to Juxtapose Mode, allowing users to unlock the door by holding their smartphone up to the sensor, typically within one second. After switching to Juxtapose Mode, the password is immediately updated. Although the door lock password does not change during Remote Mode, the lock remains securely protected for several reasons: (A) Users do not need to know or have access to the passwords, eliminating the risk of password leakage. (B) Even if an unauthorized intruder attempts to crack the door lock system, after a certain number (e.g., 100) of incorrect password entries, the system will remain locked for a set period (e.g., 10 min) and automatically switch to the Juxtapose Mode, preventing remote locking/unlocking. In this mode, passwords are frequently changed (several times per minute) to prevent further intrusion by attackers. Additionally, if the maximum number of attempted password entries is exceeded, the system can trigger a security alarm and automatically notify a private security company.

As can be seen in Fig. 4, double-length passwords can be generated by combining two passwords. However, the number of the passwords we need to retain in the password set increases in proportion to a square of the number of the passwords in the password set; thus, the number of trials required to match the passwords stored in the terminal need to be increased accordingly. For example, if 24-digit passwords are generated, 10,000 passwords need to be retained in the password set. However, the time required for 10,000 trials is negligible in comparison to the time required to crack a 24-digit password with a brute-force attack (49,000 years). Such long passwords may not be needed to protect our terminals since an operating system is locked after a certain number of trials, but these could be useful to encode the data carried via the wireless system (such as Wi-Fi) since the unlimited number of trials are allowed to decode the data in this case. IoT appliances are usually located close to each other in a house and generally remain in the same position. In such a configuration, COSMO-PASS frequently updates the 24-digit encryption keys used for communication between IoT appliances without the need for exchanging keys between these appliances, which further enhances the security of the IoT network to realize the invincible CosmoSmartLink system in smart homes.

### Performance comparison to other techniques

In order to compare the current work and other established techniques, first we review other well-spread authentication techniques. All authentication methods are currently categorized into the following three domains^33^: (A) knowledge-based authentication, (B) token-based authentication, and (C) biometric based authentication.

(A) Knowledge-based techniques, which are based on text and/or picture passwords, are the most widely used authentication techniques^34,35^. The most basic kind of example of this technique is a simple password authentication. However, due to necessity of recalling passwords, frequent password updating and keeping track of multiple passwords (particularly random sequence passwords) are difficult to manage for most individuals. According to the Zviran & Haga report, it was found that if people created a password based on words, numbers and/or symbols that have personal meaning or significance to them, more than 25% were able to recall this password correctly after 3 months; if participants created passwords by using randomly chosen characters, password recall rates dropped to almost 0%^36^. These results indicate that one meaningful password (or a few very similar meaningful passwords) tend to be reused over and over again; hence, various risks, particularly phishing, increase.

Password managers offer a solution to solve this problem. A major benefit of password managers is their ability to mitigate phishing attacks. Users do not have to actually memorize each password for each login. However, it is crucial to maintain the security of the master account and safeguard credentials such as a master password. This means that there is possibility that a single phishing attack can expose all of user’s credentials^37^. Li et al.^37^ investigated several web-based password managers and found that they are more vulnerable than local password managers from the cyberattack since sharing credentials increases the complexity of securing password managers. Li et al. pointed out four vulnerabilities in terms of bookmarklets, web, authorization and user interfaces by applying the web attacker model^38^ to five web-based password managers including LastPass^39^, RoboForm Everywhere^40^, My1login^37^, PasswordBox^41^, and NeedMy Password^42^. Moreover, they proposed several risk mitigation solutions such as establishing a form of user confirmation before sharing credentials with the website or by using Defence JavaScript^43^, a new defense based on iframes^44^, password alternatives such as Single Sign-Ons (SSOs)^45,46^.

(B) Token based authentication uses a combination of knowledge-based techniques and additional hardware such as smart cards and cellphones to enhance security^47^. Traditionally static passwords can more easily be accessed by an unauthorized intruder given enough time and attempts. The “one-time password technique” is one of the strongest token-based solutions to secure the user’s system. With this technique, the passwords are frequently updated, and used only one time. By constantly altering the password, the phishing risk can be greatly mitigated. There are several types of “one time password” methods including (A) the challenge response, (B) time synchronizing, (C) counter synchronization, and using a (D) numerical matrix Table^33^. However, this kind of scheme is not completely secure against all phishing attempts. For example, the password can possibly be stolen if an eavesdropper uses a camera to record all the screens of the system and motions of the victim^33^.

Another popular token-based technique is “multi factor authentication” (MFA). MFA is a method wherein users are required to present more than one type of evidence to authenticate on a system; hence improving the security in comparison to single password authentication. For this, MFA usually requires additional hardware such as cellphones. Niranjan reported 6 disadvantages regarding MFA^48^: (1) the management complexity for both administrators and end users. (2) it is more difficult to configure and use MFA, (3) some users don’t have the necessary devices to use other factors for authentication, (4) extra requirements for specific hardware that can introduce significant costs and administrative overheads, (5) users risk potentially being locked out of their accounts if they lose or are unable to use other factors, and (6) introduction of additional complexity into the application. Moreover, there are 2 vulnerabilities^48^: (1) processes implemented to allow users to bypass or reset MFA may be exploitable by attackers, and (2) many MFA solutions add external dependencies to systems, which can introduce security vulnerabilities or single points of failure.

(C) Biometric based authentication techniques, such as fingerprint, iris scan, or facial recognition also require additional hardware. While this type of technique provides the highest level of security, biometric based authentication techniques are not yet widely adopted due to difficulty to perform frequent and fast identification processes. The biometric systems can be expensive and sometimes unreliable^33^. The costs for the fingerprint authentication system, the vein authentication system, and the iris authentication system are respectively $3k USD-$7k USD, $5k USD -$10k USD, and $5k USD -$12k USD^49^. Biometric identification techniques such as face scanners and particularly fingerprint readers are gaining in popularity, but some of these methods are still prone to false positive and false negative identification^50^.

As described, there are several established methods for authentication. Table 3 summarizes the performance comparison between COSMO-PASS and other options. The main drawback of COSMO-PASS is applicable distance range. Due to the limited distance range of muons, unlike other systems designed to protect internet applications/websites, COSMO-PASS can only be applied to local area networks like the example shown in Fig. 12. By using the system in a hybrid mode (combining Remote Mode and Juxtapose Mode), this restriction can be slightly mitigated. However, due to the fact that the COSMOCAT unit and the CTC unit must be physically close to each other for password updates, it is difficult to universally apply this technique to authentication of various web-applications on the internet. Another caveat of COSMO-PASS is the requirement of additional hardware. However, unlike other techniques, users do not need to create/remember/store passwords with COSMO-PASS and thus, the phishing risk is ~ 0%. Similar to “MFA” and the “one-time password”, the additional security benefits of COSMO-CAT may outweigh the inherent limitations for specific purposes.

**Table 3:** Performance comparison between COSMO-PASS and other authentication techniques.

Performance	Techniques						
	Simple password^36^	Password manager^37^	SSO^51^	MFA^48^	One Time Password^33^	Biometric^33,49^	COSMO-PASS (this work)
Automatic password update	No	No	No	No	Yes	No	Yes
Necessity to memorize/store passwords (including master passwords)	Yes	Yes	Yes	Yes	No	No	No
Necessity of additional device	No	No	No	Yes	Yes	Yes	Yes
Necessity of typing/inputting passwords	Yes	Yes	Yes	Yes	Yes	No	No
Phishing risk	Yes	Yes	Yes	Yes	Yes	No	No
Distance Range	Long	Long	Long	Long	Long	Long	Short
Cost	Law	Law	Law	Law	Moderate	Expensive	Moderate

In summary, COSMO-PASS enables users to generate TRN passwords that can be shared between terminals without information exchange. The users do not have to know or to remember these frequently updated passwords and thus, there is no password leak (phishing) risk. In conclusion, it has been demonstrated that COSMO-PASS can provide a flexible and practical security solution for frequent TRN password generation in a local WSN such as a smart home. Our results indicate that: (A) the password update rate depends on the distance between the COSMOCAT unit and the CTC unit, ranging from less than 0.1 PPH to a few thousand PPH, depending on the distance (10 –500 cm) and angle (0° − 80°) formed by these units, (B) if the length of the password segment is 7 digits, the password mismatching rate was more than 1%, but if the length of the password segment is 6 digits, it was reduced to 10^− 3^. Also, unlike other authenticating techniques, (C) the COSMO-PASS technique requires the initialization process not only when first using, but also when switching from Remote Mode to Juxtapose Mode. The experimental results also indicated that at least 5 trials are required to complete this process; hence the time required for this process ranged from 0.4 s to 3.8 h depending on the distance between the COSMOCAT unit and the CTC unit. A hybrid technique for remote logins with the COSMO-PASS system was also introduced. In order to log into remote devices (located more than 2 m away), users either need to physically approach to the device and use their key detector device (such as a smartphone) to unlock the terminal, or the COSMO-PASS system needs to switch to the Remote Mode, retaining the password used for the previous authentication and temporarily bypassing the password update function, allowing the use of an older password for accessing remote devices.

## Data Availability

The datasets used and/or analyzed during the current study are available from the corresponding author on reasonable request.
